# Estimating Vehicle Fuel Consumption and Emissions Using GPS Big Data

**DOI:** 10.3390/ijerph15040566

**Published:** 2018-03-21

**Authors:** Zihan Kan, Luliang Tang, Mei-Po Kwan, Xia Zhang

**Affiliations:** 1 State Key Laboratory of Information Engineering in Surveying, Mapping and Remote Sensing, Wuhan University, 129 Luoyu Road, Wuhan 430079, China; kzh@whu.edu.cn; 2 Department of Geography & Geographic Information Science, University of Illinois at Urbana-Champaign, 1301 W Green Street, Urbana, IL 61801, USA; mpk654@gmail.com; 3 Department of Human Geography and Spatial Planning, Faculty of Geosciences, Utrecht University, P.O. Box 80125, 3508 TC Utrecht, The Netherlands; 4 School of Urban Design, Wuhan University, Wuhan 430070, China; xiazhang@whu.edu.cn

**Keywords:** fuel consumption, emissions estimation, GPS trace, big data

## Abstract

The energy consumption and emissions from vehicles adversely affect human health and urban sustainability. Analysis of GPS big data collected from vehicles can provide useful insights about the quantity and distribution of such energy consumption and emissions. Previous studies, which estimated fuel consumption/emissions from traffic based on GPS sampled data, have not sufficiently considered vehicle activities and may have led to erroneous estimations. By adopting the analytical construct of the space-time path in time geography, this study proposes methods that more accurately estimate and visualize vehicle energy consumption/emissions based on analysis of vehicles’ mobile activities (*MA*) and stationary activities (*SA*). First, we build space-time paths of individual vehicles, extract moving parameters, and identify *MA* and *SA* from each space-time path segment (STPS). Then we present an N-Dimensional framework for estimating and visualizing fuel consumption/emissions. For each STPS, fuel consumption, hot emissions, and cold start emissions are estimated based on activity type, i.e., *MA*, *SA* with engine-on and *SA* with engine-off. In the case study, fuel consumption and emissions of a single vehicle and a road network are estimated and visualized with GPS data. The estimation accuracy of the proposed approach is 88.6%. We also analyze the types of activities that produced fuel consumption on each road segment to explore the patterns and mechanisms of fuel consumption in the study area. The results not only show the effectiveness of the proposed approaches in estimating fuel consumption/emissions but also indicate their advantages for uncovering the relationships between fuel consumption and vehicles’ activities in road networks.

## 1. Introduction

As urbanization accelerates, transport-related environmental issues deteriorate. The report of Intergovernmental Panel on Climate Change (IPCC) shows that 20–30% of total greenhouse gases (GHGs) are released from urban transportation operation including passenger and freight transportation [1]. Estimating and visualizing fuel consumption and emissions from transportation provide an understanding of the energy cost and air pollution caused by travel or transportation. However, previous studies often estimated fuel consumption/emissions without considering vehicles’ activities and thus might lead to erroneous estimations. Therefore, this study proposes approaches that estimate and visualize vehicles’ fuel consumption/emissions accurately by considering vehicles’ mobile and stationary activities in a space-time-integrated framework with vehicles’ GPS trajectories data.

Traditional ways to estimate air pollution rely on air pollution monitoring stations that are located at specific sites throughout a city. Data collected by these monitoring stations can be further used to evaluate the status of atmosphere according to clean air standards and historical information. While these data are more reliable, monitoring stations are expensive to set up and maintain [2], and thus there are usually a very limited number of monitoring stations in a particular city. For the emissions and fuel consumption from the transportation sector, although it is recognized that vehicular emissions and fuel consumption play a significant role in air pollution and energy consumption, the exact volume and spatial distribution of pollution/fuel consumption of vehicles remain unknown. As the exact volume emissions and fuel consumption can only be measured with professional equipment installed on individual vehicles, such measurement can hardly be implemented in practice. Therefore, emissions and fuel consumption estimation approaches have been widely investigated in past decades.

At early stages, some researchers estimated fuel consumption/emissions from aggregate fuel-used data at a large spatiotemporal scale [3]. However, these studies only provided a rough estimation due to a lack of information about vehicle technology and moving parameters. In past decades, energy/emission estimation models, such as the U.S. EPA’s MOBILE and MOVES models, European Commission’s COPERT model, California’s EMFAC and IVE model, have been extensively developed [4,5,6,7,8]. In these models, vehicle technology data and moving parameters are necessary for estimating fuel consumption/emissions. In order to collect these data and estimate fuel consumption/emissions with the estimation models, surveys and sensors that are installed in some segments of a road such as loop detectors [9] and video cameras [10] have been used in the literature. However, details of vehicles’ driving parameters are absent in survey data and loop detector data. Large-scale survey data can only provide vehicle technology and rough driving speeds in a city or a nation, and loop detector data only contains traffic information collected at specific locations on roads that are not representative enough for the driving parameters over larger road segments. Therefore, survey data and loop detector data can only be employed to estimate fuel consumption/emissions at a coarse spatial resolution, or as a supplement to other estimation approaches.

The rapid development of data collection, storage, and networking have created an environment with big data infiltrating many aspects of society and technology. As an important component of big data, GPS trajectory data are widely used due to their large coverage, good continuity, low cost, as well as rich information about vehicles’ movements. Vehicle trajectories contain rich information about vehicles’ driving modes and traffic states, which could be used to fit emission models to obtain more accurate emissions estimations. Early studies used vehicle trajectory data to estimate macroscopic pollutant emissions at the city scale [11]. In recent years, some researchers proposed emissions estimation methods that used GPS tracks of vehicles [2,12,13,14,15]. These methods quantified fuel consumption and emissions using emission models based on the premise that the amount of pollution emitted by a vehicle mainly depends on load and moving parameters [16]. For example, Sun et al. [13] and Shang et al. [14] reconstructed traffic volume from GPS data so that traffic-related emissions can be estimated based on the trajectories of sampled vehicles and the developed estimation model.

Among all kinds of vehicles’ trajectory data, taxis trajectory data have become a popular data source for traffic monitoring and fuel consumption/emissions estimation, as taxis are important part of urban transportation systems and account for a large share of urban traffic flows. Gühnemann et al. [11] estimated traffic NOx emissions using GPS data from a fleet of taxis using an average speed-dependent estimation model. Based on the COPERT model, Shang et al. [14] analyzed the patterns of fuel consumption/emissions in Beijing using taxi GPS trajectory data. Luo et al. [17] analyzed the spatial-temporal patterns of taxis’ fuel consumption/emissions as well as the relationships between taxis’ travel patterns and fuel consumption/emissions. These studies estimated fuel consumption/ emissions with low-sampling GPS data of 30–60 seconds. They adopted macroscopic estimation models that take variables such as vehicle category constitution, fuel parameters, emission legislation, and average speed of vehicles into consideration but did not need detailed parameters of vehicles’ driving modes such as accelerations in the estimation.

In order to distinguish the different driving modes of vehicles (acceleration, deceleration, idling and cruising) and estimate the fuel consumption/emissions of vehicles in a more fine-grained way, some researchers adopted high-resolution GPS data and microscopic estimation models in their work. Nikoleris et al. [18] proposed a detailed estimation of fuel consumption and emissions using aircraft position data. Engine emissions inventories which provide fuel flows and emission indices as a function of engine thrust were used in their study. Zhao et al. [15] estimated CO_2_ emissions using taxi GPS data and analyzed the relationships between CO_2_ emissions and trip purposes. The estimations in their study was based on a microscopic model that considers instantaneous driving modes of vehicles, whereas the GPS data in their study were sampled at 60 s, indicating that the data could not reflect vehicles’ instantaneous condition. Sun et al. [13] estimated emissions of vehicles under different driving modes (e.g., acceleration, idle, cruise and deceleration) with variables of second-by-second speed profiles. Nyhan et al. [2] estimated taxi emissions in Singapore based on the microscopic emission model proposed by Osorio and Nanduri [19], in which instantaneous speed, instantaneous acceleration, vehicle type and fuel type are all needed in the estimation. High-frequency GPS data (less than 5 s) were used in the study to obtain the instantaneous moving parameters. Although the parameters of the driving modes of vehicles contained in high-resolution GPS data result in more-accurate estimations, high-frequency GPS data are not used in many studies due to the additional cost in data collection and storage, as well as the complexity and computational inefficiency in estimation. Despite the lower-resolution of macroscopic models, the analytical structural information they provide can contribute to enhancing the computational efficiency of fuel consumption/emissions estimations [19].

While driving mode parameters such as acceleration and deceleration can be obtained with high-resolution GPS data, existing studies estimating fuel consumption/emissions with GPS trajectory data lack analysis of vehicles’ stationary activities. In a movement path, an activity conducted by a vehicle from one location to another location is a mobile activity (*MA*), and an activity conducted in a fixed location is a stationary activity (*SA*). An *SA* of a vehicle may occur under conditions of engine-on or engine-off. For an *SA* with engine-on, vehicles consume fuel and release emissions. Estimating factors of fuel consumption/emissions of *SA*s with engine-on are different with *MA*s. For an *SA* with engine-off, vehicles do not consume fuel or release emissions. However, additional cold start emissions are released when a vehicle restarts. Previous studies did not analyze different types of *SA*s in vehicle trajectories. As a result, hot emissions generated when the vehicle’s engine is running and cold start emissions released during the engine’s warming-up phase cannot be distinguished. Research results show that about 20% of the total emissions from vehicles are cold start emissions [20]. Therefore, the distinction between hot emissions and cold start emissions is important due to the substantial differences in vehicular emission performance during those two phases, and a different approach is required to estimate over-emissions during the engine’s warming-up period.

Table 1 illustrates the characteristics of data and models in the aforementioned studies and this study. For each study, Table 1 lists the resolution of GPS trajectory data, the emissions model used in each study, whether driving modes and stationary activities are considered, and whether hot emissions and cold start emissions are distinguished. For the emission models in the table, the basic variables of emissions and fuel consumption estimation include vehicle category, fuel type and travel speed of vehicles. While parameters of road condition such as road type and slope are available in the COPERT model that is used in studies [14,17], and the CMEM model used in study [13], such parameters have not been widely used for estimating emissions and fuel consumption in practice due to the lack of data. This study estimates vehicles’ consumption/emissions based on the COPERT model, which is a typical mathematical model. Based on distinguishing vehicle categories, fuel types and other parameters, the COPERT model determines the emission of different pollutants and consumption by performing regression analysis for speeds of vehicles and volume of emissions/consumption. As the operating conditions and engine technologies of the test vehicles in the COPERT model are similar to that of the experiments in this study, the estimated emissions/consumption are considered closer to the true value. The key estimation parameters in the COPERT model include constitution of fleet, average speed, average mileage, fuel parameters, load and slope, among which the default value of average mileage is provided in case that the parameter is absent in estimation. Therefore, based on the determination of all the parameters in the COPERT model, the emissions/consumption factors are modeled as functions of average speed of vehicles. In contrast, the microscopic estimation models in studies [2,13,18] can simulate the instantaneous moving conditions of vehicles and hence generate more accurate estimations. In that case, instantaneous acceleration is a better parameter to model the engines’ thrust compared with instantaneous speed and average speed. The parameters of vehicle engines such as type, age or other performance parameters are very important in some microscopic models such as the CMEM model (in study [13]) which investigate the physical relationships between the instantaneous operating states of vehicles and instantaneous emissions/consumption. These estimation models can simulate the emissions/consumption accurately while they have not been widely used in large-scale emission/consumption estimations as engine parameters are hard to acquire.

Table 1 shows that recent studies that used low-resolution GPS data included average moving parameters such as average speed into macroscopic estimation models to estimate fuel consumption/emissions of a road segment. However, it is difficult to identify driving modes such as acceleration, idling, deceleration and cruising in these studies due to the low resolution of the data. While research conducted with high-resolution GPS data includes driving mode analysis, stationary activities analysis is absent in these studies. As a result, different phases of emissions over a particular driving cycle are not distinguished in recent studies with both low-resolution and high-resolution data. Therefore, the purpose of this article is to estimate vehicles’ fuel consumption/emissions more accurately by taking into account vehicles’ activity types.

In order to differentiate cold start emissions and hot emissions in a vehicle’s driving cycle, *MA*s, *SA*s with engine-on and *SA*s with engine-off need to be analyzed first. In this study, we analyze different types of vehicle activities based on the space-time path of individual vehicles. The space-time path was developed by Hägerstrand [21] and his colleagues at Lund University as a component of the time-geographic framework, which is a powerful approach for analyzing movement patterns of individuals in space and time. Originally, time geography was used mainly to investigate the movement and activity-travel patterns of humans [22,23,24,25,26]. It was later applied to transportation networks [27,28,29]. One of the core problems in time geography is to visually represent different elements through a 3-D space-time framework [23,30]. A space-time path portrays the trajectory of an object in a 3-D orthogonal system consisting of two spatial dimensions (a plane) and a vertical temporal dimension. Not only people’s activity patterns [23,24,31,32] but also moving parameters in one’s trajectory [25] can be represented by space-time paths.

This study proposes approaches that accurately estimate and visualize vehicles’ energy consumption/emissions based on analysis of vehicles’ mobile activities (*MA*) and stationary activities (*SA*). Different phases of emissions over a particular driving cycle, i.e., hot emissions and cold start emissions are estimated based on activity analysis. In the case study, fuel consumption and emissions of a single vehicle and a road network are estimated and visualized with GPS trajectory data. We also analyze the types of activities that produced fuel consumption on each road segment to explore the patterns and mechanisms of fuel consumption in the study area. To our best knowledge, this is the first study to estimate different phases of taxi emissions with GPS trajectory data and the first study to distinguish stationary and moving activities in fuel consumption/emissions estimation. 

## 2. Materials and Methods

### 2.1. Data

This study first estimates fuel consumption and emissions for an experimental vehicle and verify the estimating accuracy of fuel consumption, then estimates fuel consumption and emissions for an experimental area with taxis’ GPS trace data.

#### 2.1.1. A Single Trajectory with Real Fuel Consumption

We first estimate the fuel consumption and emissions for a gasoline vehicle with its GPS trajectories. The GPS trajectory data were collected from an experimental vehicle of our project team, and its real fuel consumption was recorded during the time period of experiment (from 23 March to 25 March 2016) to validate the estimation methods. The GPS data have an approximate sampling interval of 10 s, a minimum sampling interval of 6 s, a maximum sampling interval of 16 s, an average sampling interval of 10.56 s and a standard deviation of the sampling interval of 0.52 s. A description of the data is shown in Table 2, where “VID” represents the vehicle’s ID, “Time” denotes the sampling timestamp when a record was generated, “Longitude” and “Latitude” are the geographic location of the vehicle at the timestamp. In order to estimate the fuel consumption and emissions more accurately, the temperatures are needed, which are 12 °C, 9 °C and 10 °C on 23–25 March, respectively.

**Figure 1:**
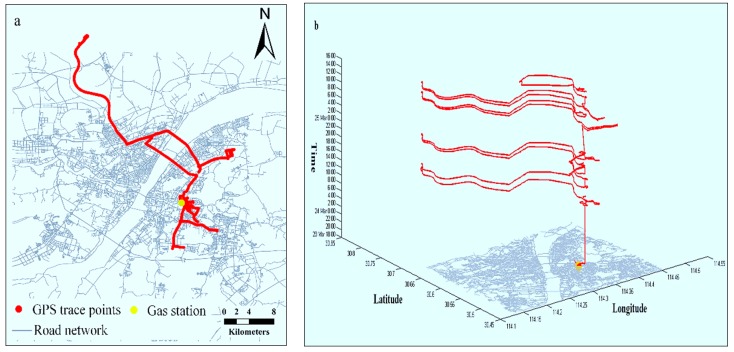
The vehicle’s GPS trace and space-time path. (**a**) Shows the GPS traces of the vehicle and (**b**) Describes the space-time path built from GPS trace.

We recorded the real fuel consumption for verifying the estimating accuracy of the proposed approach. The detailed information of the vehicle model, fuel, and real fuel consumption are shown in Table 3. The GPS data and space-time paths of the vehicle are shown in Figure 1.

#### 2.1.2. The Experimental Network Area and Taxi GPS Dataset

Second, we use GPS data to estimate the fuel consumption an emissions for an experimental area in Wuhan, China. The GPS data were obtained from the Wuhan Transportation Bureau, which were collected from 6658 taxis operating in the urban area of Wuhan on 6 May 2015 (Friday). The taxis account for about 44% of all the taxis in the urban area. 

**Figure 2:**
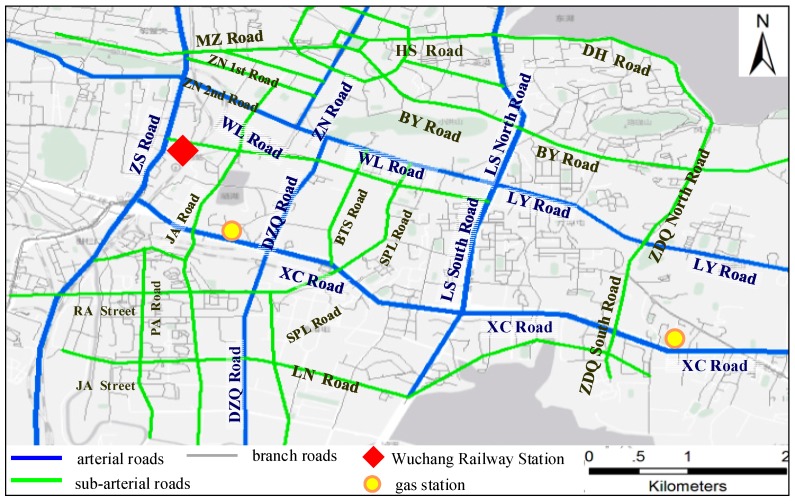
Road network in the experimental area.

The taxi GPS trajectories were sampled at an approximate time interval of 60 s (the minimum sampling interval is 11 s, the maximum sampling interval is 121 s, the average sampling interval is 62.9 s and the standard deviation of the sampling interval is 20.5 s), with a position accuracy of approximately 15 m. A description of the taxi GPS data is shown in Table 4, where “VID” stands for the taxi’s ID, “Time” is the sampling time when a record was generated, “Longitude” and “Latitude” are the geographic location of the taxi, and “Direction” represents the taxi’s driving direction, “Speed” provides the instantaneous velocity (km/h), “Status” represents the taxi’s passenger status (1 denotes an occupied status and 0 indicates a vacant status). The temperature on 6 May 2015 is 21.5 °C. The road network in the study area is shown in Figure 2, in which the road names are abbreviated.

### 2.2. Methods

This section describes fuel consumption/emissions estimation based on space-time path segment (STPS). We present an N-dimensional framework for estimating and visualizing fuel consumption/emissions of both an individual vehicle and a network area. Hot emissions and cold start emissions of each STPS are also distinguished based on activity types.

#### 2.2.1. Building Space-Time Path and Extracting Moving Parameters

**Figure 3:**
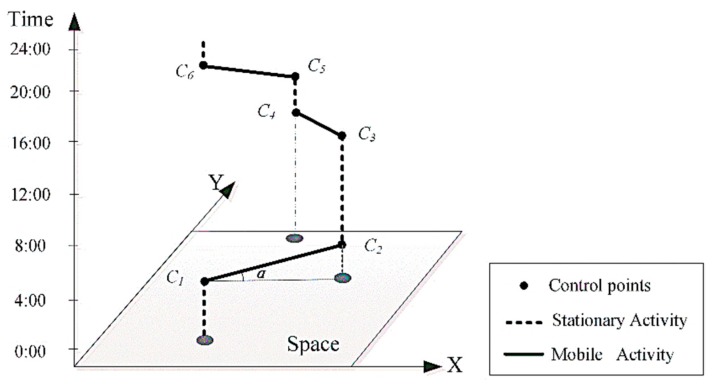
3-D Space–time coordinate and individual space-time path.

Based on the vehicles’ GPS data, this study first builds space-time paths for individual vehicles according to the locations and timestamp of each GPS track point. A space-time path depicts the movements of individuals in both space and time. Each space-time path comprises a series of space-time path segments (STPS), which are the line segments between consecutive track points. In this section, we build space-time path of an individual vehicle and extract moving parameters of each STPS. The space-time path of an individual in space-time coordinate is as Figure 3 shows.

In Figure 3, the space-time path *P^m^* for an individual *m* consists of a sequence of control points and a corresponding sequence of STPSs connecting the control points [33]. The control points are a finite list of observations in chronological order, normally collected by location sensors such as GPS devices. Each control point, *c_i_*, consists of a tuple:

*c_i_* = <*Loc_i_*, *t_i_*> (1)

where *Loc_i_* is the location and *t_i_* the time stamp. The STPS *s_i_* between adjacent control points *c_i_* and *c_i_*_+1_ is a straight line segment:

*s_i_* = <*c_i_*, *c_i_*_+1_> (2)

It is assumed that the individual is moving at a constant speed in each STPS [33], based on which the speed of the STPS *s_i_* is:

(3) $$v_{i}=\frac{\left\|s_{i}\right\|}{t_{i+1}-t_{i}}$$

where ‖*s_i_*‖ is the length of STPS *s_i_*. If the instantaneous velocity of each control point *v’* is recorded, average acceleration *a_i_* of an STPS *s_i_* can be calculated by:

(4) $$a_{i}=\frac{v_{i+1}^{\prime }-v_{i}^{\prime }}{t_{i+1}-t_{i}}$$

In time geography, each STPS represents at least one activity, which consists of two adjacent GPS track points. As is shown in Figure 3, an activity conducted from one location to another location is a mobile activity (*MA*), depicted by a tilted line segment (the solid line in Figure 3), and an activity conducted at a fixed location is a stationary activity (*SA*), depicted by a vertical line segment (dashed line in Figure 3). In this article, *MA* and *SA* can be defined by:

*MA* = {(*Loc*(*S*), *T_S_*), (*Loc*(*E*), *T_E_*), || *Loc*(*E*) − *Loc*(*S*)| ≥ *δ*} (5)

*SA* = {(*Loc*(*S*),*T_S_*), (*Loc*(*E*),*T_E_*), || *Loc*(*E*) − *Loc*(*S*)| < *δ*} (6)

where *Loc*(*S*) and *Loc*(*E*) are locations of start and end points of activities, *T_S_* and *T_E_* the start and end times. In Equations (5) and (6), *Loc*(*S*), *Loc*(*E*), *T_S_* and *T_E_* can be obtained through the coordinates and timestamp of the adjacent GPS track points. Besides, the threshold *δ* can be set according to the GPS positional error (approximately 15 meters in this study). For each activity, if the distance between the adjacent GPS track points is smaller than *δ*, there is a stationary activity (*SA*); if the distance between the adjacent GPS track points is larger than *δ*, there is a mobile activity (*MA*). The value of *δ* would impact the classification of activity and the estimation of cold start emissions because cold start emissions are closely related to vehicles’ stop behaviors. Setting the value of *δ* too high would lead to misidentifications of *MA*s as *SA*s, resulting in overestimations of cold start emissions. Setting the value of *δ* too low would lead to misidentifications of *SA*s as *MA*s, resulting in underestimations of cold start emissions.

#### 2.2.2. An N-Dimensional Representation for Fuel Consumption/Emissions Based on Space-Time Path

Based on individual space-time path, we present an N-dimensional representation for visually estimating fuel consumption/emissions and representing different multi-dimensional information based on STPS. Each STPS contains multi-dimensional information of individuals including space and time, fuel consumption/emissions, moving parameters (e.g., speed, acceleration) and other individual attributes (e.g., human or vehicle, age, gender). Therefore, in the N-dimensional representation describing N attributes of individuals, a space-time path consisting of *M* STPS can be described as a M×N matrix, as Equation (7) shows:

(7) $$P={\left[\begin{array}{c} S_{1}.Time\\ S_{2}.Time\\ \ldots \\ S_{M}.Time \end{array}\begin{array}{cccc} S_{1}.Location & S_{1}.speed & \ldots & S_{1}.consumption\\ S_{2}.Location & S_{2}.speed & \ldots & S_{2}.consumption\\ \ldots & & \ldots & \ldots \\ S_{M}.Location & S_{M}.speed & \ldots & S_{M}.consumption \end{array}\begin{array}{c} S_{1}.emissions\\ S_{2}.emissions\\ \ldots \\ S_{M}.emissions \end{array}\right]}_{M\times N}$$

**Figure 4:**
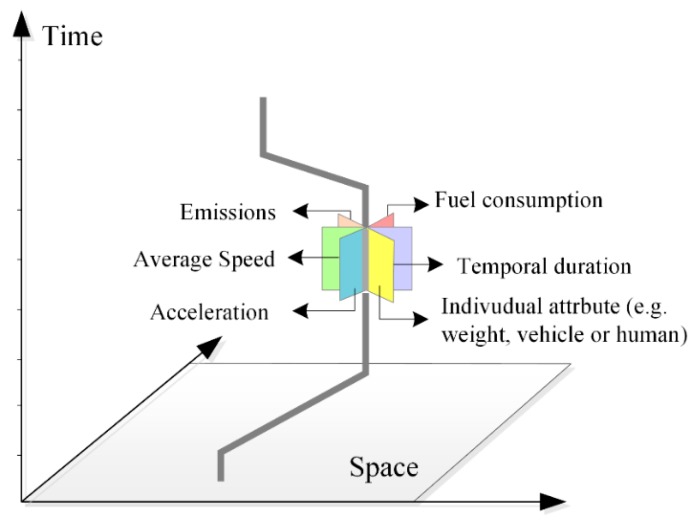
N-dimensional model of individual space-time path.

The visualizing framework of the N-Dimensional model is shown in Figure 4, where each STPS is attached with different dimensions of information represented by rectangles with different colors.

**Figure 5:**
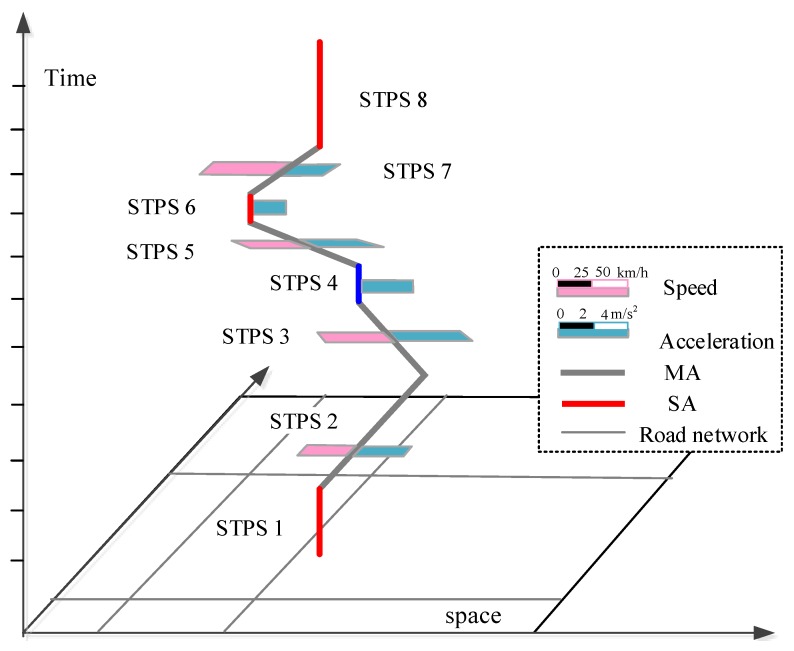
N-Dimensional representation of moving parameters.

The N-dimensional representation of moving parameters is shown in Figure 5. In the representation, different dimensional information of each STPS is represented by attached rectangles with different colors. In the representation of acceleration, there is no speed but acceleration for STPSs 4 and 6 because they are SAs with engine-on, and there are no speed and acceleration dimensions for STPSs 1 and 8 because they are SAs with engine-off.

#### 2.2.3. Estimating Fuel Consumption/Emissions Based on Individual Space-Time Path

In this section, we estimate fuel consumption and emissions on an STPS basis. Hot emissions and cold start emissions of each STPS are also distinguished based on the determination of vehicles’ activity types.

**Figure 6:**
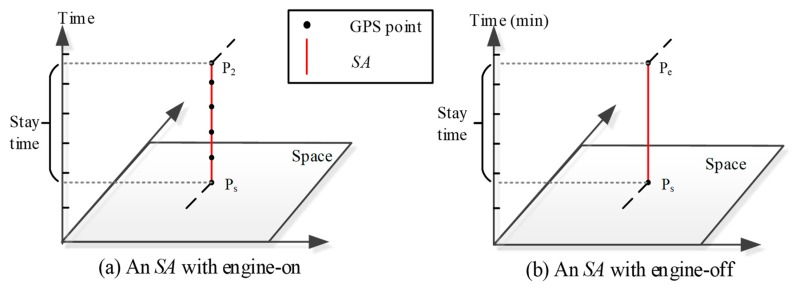
Vehicles’ *SA* with (**a**) Engine-on and (**b**) Engine-off.

Firstly, we determine the operating conditions of vehicles. Section 2.2.1 demonstrates that each STPS represents either a mobile activity (*MA*) or a stationary activity (*SA*). For an *MA* STPS, fuel consumption and emissions can be estimated according to moving parameters such as average speed. While operating conditions of *SA* STPS need to be further distinguished in fuel consumption/emissions estimation, as an *SA* may happen with the engine-on or with engine-off. In both situations, the average speeds of an STPS are zero. However, for *SA*s with engine-off, vehicles do not consume fuel or release emissions, but for *SA*s with engine-on, vehicles need to consume fuel and thus release emissions to keep the engines running. Therefore, this article infers vehicles’ operating conditions from stay time of an *SA* and GPS sampling frequency, as Figure 6 shows. Figure 6a represents a *SA* with engine-on, in which the locations of the vehicle is recorded at a sampled frequency during the stay time. Figure 6b depicts an *SA* with engine-off, in which there are no GPS points recorded during the stay time of the *SA*.

Secondly, we estimate fuel consumption and emissions of *MA*s based on COPERT model [4]. This article adopts COPERT model in the estimation of *MA*s because the vehicles that the data are collected from in the case study follow European-3 emission standard. We estimate the consumption and emissions from average speeds and lengths of each STPS. We first estimate fuel consumption for each STPS, then estimate hot emissions and cold start emissions for each STPS.

- Fuel Consumption Estimation

In COPERT model, the fuel consumption for *MAs* of gasoline passenger cars with a capacity of 1.4 L–2.0 L is estimated according to Equation (8), where *FC* is the fuel consumption factor (g/km) of each STPS, and *V* is the average speed of each STPS:

*FC_MA_* = (217 + 0.253*V* + 0.00965*V*^2^)/(1 + 0.096*V* − 0.000421*V*^2^) (8)

Because COPERT model doesn’t contain the emissions factor of *SA*s with engine-on, the fuel consumption for *SA*s with engine-on is estimated based on four-mode elemental fuel model [34,35], as Equation (9) shows, where the unit for *FC* is milliliters, and *T* is stay time(seconds) of an *SA*:

*FC_SA_* = 0.361*T (9)

Therefore, fuel consumption (FC) for the space-time path of a vehicle that includes fuel consumption for both *MA*s and *SA*s with engine-on can be calculated based on Equations (8) and (9):

*FC* = FC_MA_*Dist + FC_SA_*σ (10)

where *Dist* is the driving distance for a vehicle and δ is the density of the fuel. For gasoline, σ = 0.77 g/mL.

- Emissions Estimation

For *SA*s with engine-off, there are no emissions. For other activities, the total emissions are composed of three components, which are:

*E_TOTAL_* = *E_HOT_* + *E_COLD_* + *E_EVAP_* (11)

In Equation (11), *E_TOTAL_* is total emissions of a certain pollutant. *E_HOT_* refers to the emissions under the condition of thermal stability of the engine. *E_COLD_* is the emission during starting process of the engine. *E_EVAP_* is the emission caused by temperature changes. This study estimates hot emissions and cold start emissions because of their prominence in the total emissions.

**Figure 7:**
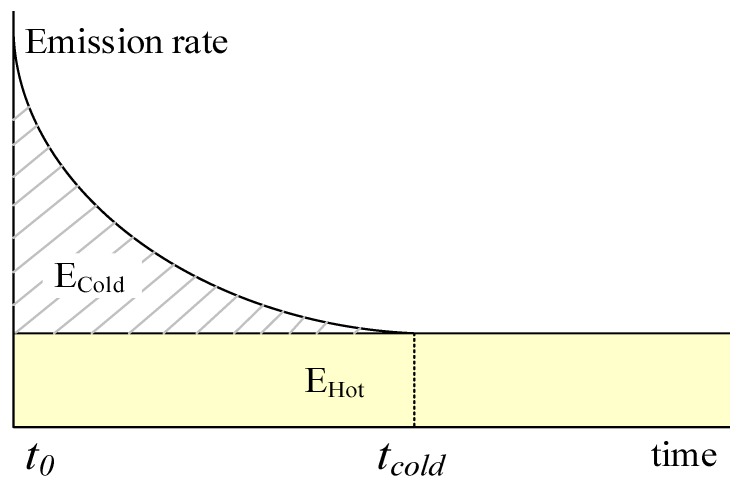
Hot and cold start emissions with time.

Figure 7 illustrates hot and cold start emissions as a function of time after a vehicle restart with its engine and catalyst reaching ambient temperature. Cold start emissions are initially high but decrease as the temperatures of engine and catalyst increase, followed by a stable phase when the normal operational temperatures have been reached. The duration of the first cold-start phase is signified by *t_cold_*, and the emission during cold-start phase is given by *E_cold_*, as shaded area shows. Whereas hot emission rate keeps stable with time, and the emission during thermally stable operation is given by *E_hot_*.

(1) Hot Emission

Hot emissions for *MAs* are determined by the average-speed-dependent baseline emission factor and vehicle’s mileage correction and fuel effect correction. Generally, the hot emission factors after legislation Euro-1 is represented by Equation (12):

*EF_MA_* = (a + c*V* + e*V*^2^)/(1 + b*V* + d*V*^2^) (12)

where *EF_MA_* is the hot emission factor for an *MA*, denoted by the mass per kilometer (g/km), *V* the average speed, and a-e parameters in emission determination. For example, for gasoline passenger cars with a capacity of 1.4 L–2.0 L under Euro-3 standards, the parameters a-e for CO, NO_x_ and Hydrocarbon are shown in Table 5.

For *SAs* with engine-on, the emissions is estimated as Equation (13) shows [34,35], where the unit for *EF* is milliliters, and *T* is stay time(seconds) of an *SA* for CO, NOx and Hydrocarbon, α equals to 13.889, 0.566 and 2.222, respectively:

*EF_SA_* = α*T (13)

In this way, emissions for the space-time path of a vehicle that includes fuel consumption for both *MA*s and *SA*s with engine-on can be calculated based on Equations (12) and (13) and as shown in Equation (14), where *Dist* is the driving distance for a vehicle and *δ* is the density of the fuel. For gasoline, σ =0.77 g/mL:

*EF* = EF_MA_*Dist + EF_SA_*σ (14)

(2) Cold Start Emissions

Cold start emissions are produced when a vehicle restarts and depend on parameters such as engine and catalyst temperature when the vehicle restart, ambient temperature, moving speed, and distance of the new trip. The parameter of engine/catalyst temperature when a vehicle starts is important in the cold start emission estimation but is hard to obtain. However, the engine/catalyst temperature can be inferred according to ambient temperature and the vehicle’s parking time with engine-off. Therefore, we adopt a method which models cold start emissions as a function of ambient temperature *T*, average speed *V*, traveled distance *d* and parking duration *t* [36]. The parking duration is the stay time of *SA*s with engine-off in this study.

In the model, the cold start emission (gram) per start can be calculated by:

*E_cold_*(*T*, *V*, *δ*, *t*) = *f*(*T*, *V*)*h(*ş*)*g(*t*) (15)

where *ş* is dimensionless distance. The functions *f*(*T*, *V*), *h*(*δ*) and *g*(*t*) for CO, HC and NOx are given in Table 6.

**Figure 8:**
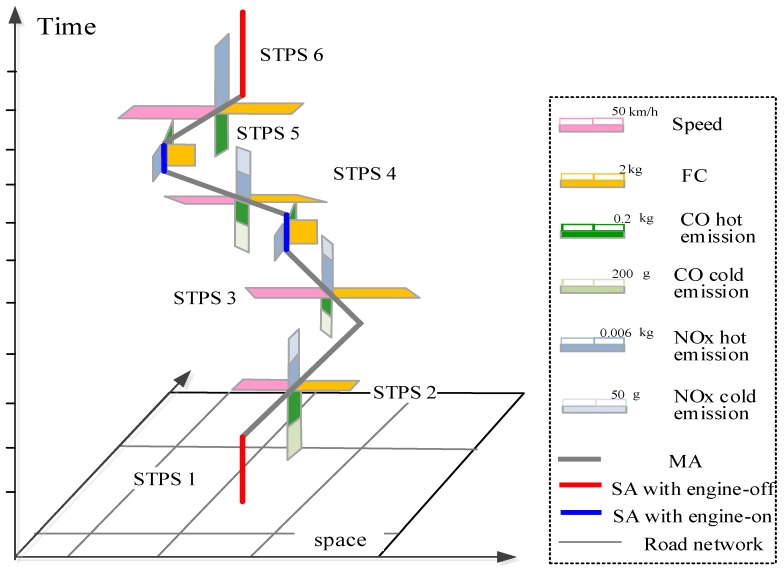
Representation of multi-dimensional information in N-Dimensional framework.

Finally, fuel consumption (*FC*) and different kinds of emissions can be estimated according to the Equations (8)–(15). Based on the estimation, we represent multi-dimensional information of each STPS using the proposed N-dimensional model, as Figure 8 shows.

In Figure 8, speed, fuel consumption, CO and NOx emissions of each STPS are represented by attached rectangles with different colors. STPSs representing *MA*s and *SA*s with engine-on are attached with fuel consumption and emissions, but there is no fuel consumption or emissions on *SA*s with engine-off. In the space-time path, the rectangles for cold start emissions are only attached in STPSs 2–4, because cold start emissions are released after a vehicle’s stop with engine-off and restart, and last for a period. After the engine has got its normal operating temperature, there are only hot emissions.

#### 2.2.4. Estimating Fuel Consumption/Emissions for Road Network

After get the fuel consumption and emissions for individual space-time path, we estimate fuel consumption/emissions for road network based on STPS. For a time interval [*ts*, *te*], fuel consumption/emissions volume for a road is estimated according to Equation (16):

(16) $$Vol_{j}^{k}(ts,te)=\sum_{i=1}^{N}\int_{ts}^{te}[EF_{i}^{k}(v)\cdot (P_{i}(t)\cap R_{j})]dt$$

where *k* is the category of fuel consumption/emissions, *j* is the *j*^th^ road in the study area, $Vol_{j}^{k}\left(ts,te\right)$ is the fuel consumption/emissions volume for a road *j* in a time period [*ts*, *te*]. *N* denotes the number of space-time paths in the experimental area. *P_i_*(*t*) is the space-time path of an individual *i*, because the locations of an individual are considered as a function of time. $EF_{i}^{k}\left(v\right)$ is the fuel consumption/emission factor (kg/km) for a fuel consumption/emission *k* and a space-time path *i*. $P_{i}\left(t\right)\cap R_{j}$ represent the intersection of a space-time path *i* and road *j*. $EF_{i}^{k}\left(v\right)\cdot \left(P_{i}\left(t\right)\cap R_{j}\right)$ indicates the volume of fuel consumption/emissions *k* of an individual *i* on road *j* at time instant *t*. Therefore, Equation (16) represents the volume of fuel consumption/emissions *k* for a road in a time interval [ts, te].

**Figure 9:**
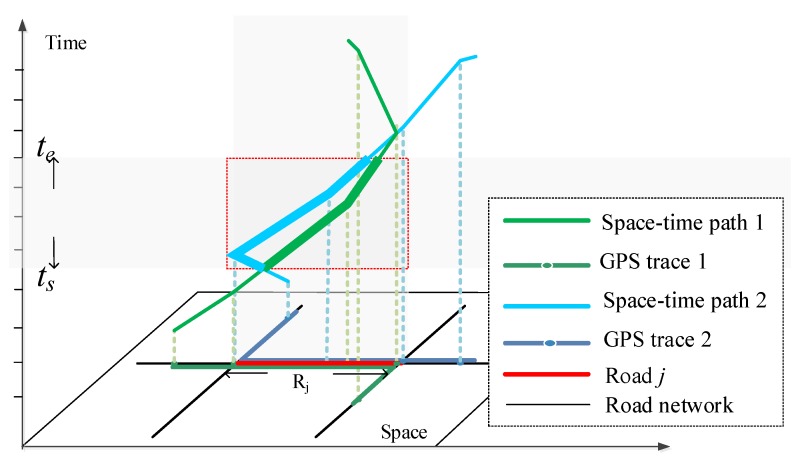
Fuel consumption/emissions estimation for road network.

Figure 9 illustrates the visual estimation and representation of fuel consumption/emissions for a single road and road network. STPSs during the time interval [ts, te] and matched to Road *j* are firstly identified. Then for each STPS, we estimate fuel consumption/emissions, and lastly we add up the fuel consumption/emissions of all STPSs to obtain fuel consumption/emissions for each road in the road network area.

## 3. Results

### 3.1. Estimating Fuel Consumption and Emissions for a Single Trace

First, we determine the activity types (*SA* or *MA*), calculate the average speed of each STPS, and get 3975 *MA*s and 2528 *SA*s. Then we analyze the running condition for each *SA* and obtain 357 *SA*s with engine-on and 2171 *SA*s with engine-off. After that, fuel consumption of *MA*s and *SA*s with engine-on are estimated based on the COPERT model. As a result, fuel consumption of the whole space-time path is calculated based on the fuel consumption of *MA*s and *SA*s with engine-on. The estimation results are compared with the estimated result from the average speed of the vehicle’s trajectories [11] and results from the average speed between adjacent GPS points [13], as shown in Table 7.

Table 7 shows the comparison results of three estimating approaches using GPS trajectories for estimating fuel consumption of the experimental vehicle. The first approach [11] uses the average speed of a whole trajectory and the total travel distance for estimating fuel consumption. This approach overestimates the fuel consumption (57.25 kg) because it takes all types of activities in a trajectory into estimation including *SA*s with engine-off, which actually produce no fuel consumption or emissions.

As a result, the first approach gets a relatively low accuracy of 71.02%. In the second approach [13], speed and distance of each pair of adjacent GPS points are calculated, according to which fuel consumption is estimated. The second approach takes the changes of speeds in the trajectory into consideration, and hence gets a better result than the first approach with an accuracy of 78.2%. However, the activity types are not distinguished in the second approach. For example, a parked vehicle (with engine-off) or an idling vehicle (with engine-on) both have speeds of zero, but they have different fuel consumptions. The second approach takes all types of activities with speed of zero as *SA*s with engine-off and with no fuel consumption. The estimating result shows that it underestimates the fuel consumption because of undifferentiated estimation of activity types. In contrast, the STPS-based approach in this study estimates fuel consumption based on activity types of each STPS. We calculate the speed and distance of each STPS, determine the activity types of each STPS (*MA*, *SA* with engine-on or *SA* with engine-off), and estimate the fuel consumption of each *MA* and *SA* with engine-on. The estimation results illustrate that accuracy is significantly improved (88.6%) when compared with the other two estimation approaches (71.02% and 78.2%), which indicates that the STPS-based approach this study develops can better restore the running condition of vehicles.

Nevertheless, there is also a gap between the estimations of this study and the real value. There are probably two reasons for the error. First, since any estimation model cannot fully simulate all the emission/consumption-related factors—such as the continuous conditions of vehicle engines, road conditions, ambient environments, and driving behaviours—all estimation models can only estimate the results as close to the real value as possible rather than generate completely accurate estimations. Second, this study estimates emission/fuel consumption based on the COPERT model, which is a mathematical model that determines the emission of different pollutants and consumption by performing regression analysis for speeds of different types of vehicles and the volume of emissions/consumption. Because the COPERT model takes the average speed as the key parameter in estimation without simulating the instantaneous conditions of vehicles, the estimated results would have errors when compared to the real values. For example, within the time period of a sampling interval, a vehicle may maintain a constant speed or it may accelerate then decelerate. In both cases the average speeds are the same but the emissions and fuel consumption are different. Therefore, using high-resolution data with small sampling intervals can reduce the error because fine sampling can reduce the uncertainty of vehicles’ driving modes within the sampling interval. Since the instantaneous parameters are not available in many practical cases, this study develops an estimating method that can be applied to large-scale GPS datasets and can be used to estimate emissions/fuel consumption more accurately than other average-speed-based methods by considering different types of activities.

**Figure 10:**
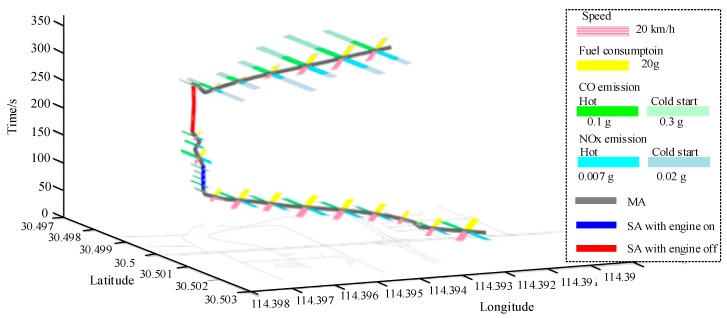
N-dimensional representation of a part of space-time path in a single trace.

Figure 10 shows the N-dimensional representation of a part of space-time path of the experimental vehicle’s trace. Two *SA*s are identified in the space-time path. The first *SA* (the blue line) is an *SA* with engine-on, so fuel consumption and emissions are estimated for it. Whereas the second *SA* (the red line) is an SA with engine-off, so there is no fuel consumption and emissions. In addition, from Section 2.2.3 we know that cold start emissions are produced after a vehicle’s engine shuts down and restart. Therefore, cold start emissions are estimated for the STPS after the *SA* with engine-off, according to Equation (15).

### 3.2. Estimating and Analyzing Fuel Consumption/Emissions for a Network Area

**Figure 11:**
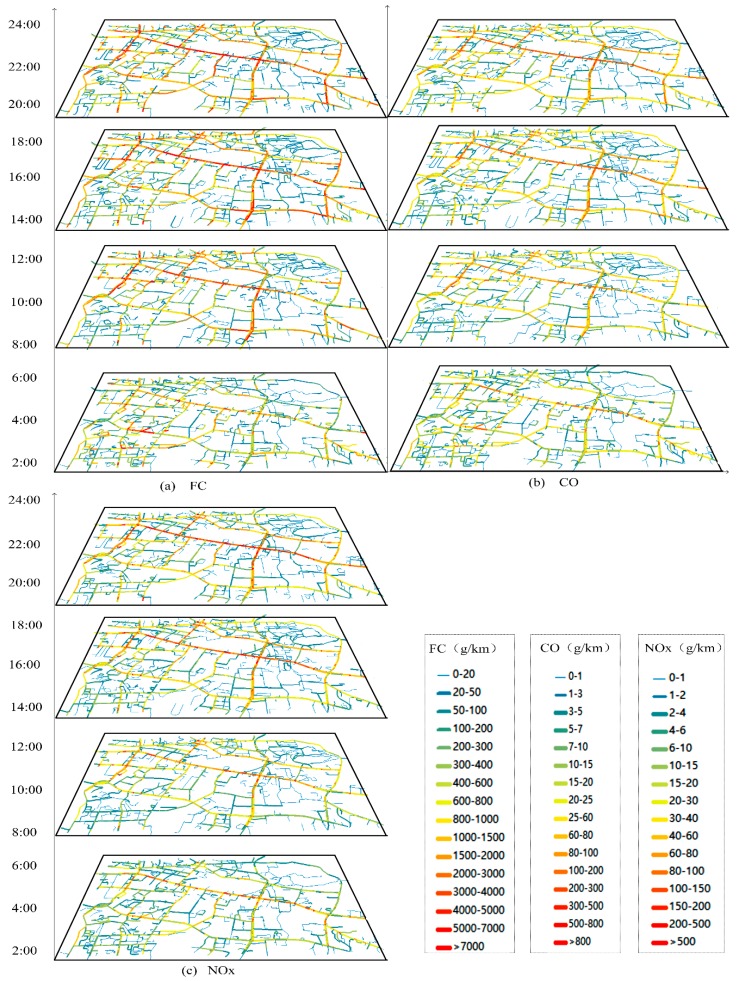
Space-time visualization of fuel consumption/emissions estimation. (**a**) Fuel Consumption, (**b**) is CO emission and (**c**) is NOx emission.

Using the taxi GPS dataset, we firstly match the GPS points to the road network, and build space-time paths for all traces according to the recorded timestamps and locations. With the GPS points matched to the road network, we can restore the routes of the taxis and obtain more accurate distances and speeds. Then *MA*s, *SA*s with engine-on and *SA*s with engine-off are distinguished based on average speed and stay time of each STPS. As a result, fuel consumption, hot emissions and cold start emissions of each STPS are estimated based on Equations (8)–(15). In this study, the fuel consumption and emissions for vehicles of both 1.4–2.0 L and 3.0 L are estimated based on the COPERT model, in which there are different sets of parameters for different vehicle types. Since the average speed is the key parameter in the estimation, the model is applicable to different types of vehicles. Finally, the whole fuel consumption, CO and NOx emissions for each road and for different time periods are obtained. Figure 11a–c shows the visualization results of fuel consumption/emissions estimation in the study area during periods of 2:00–4:00, 8:00–10:00, 14:00–16:00 and 20:00–22:00.

Obvious spatial and temporal patterns of fuel consumption and emissions are recognized with the big data analysis, as Figure 11 shows. First, the volume of fuel consumption is far more than CO and NOx emissions, and NOx emissions are the least. During 2:00–4:00, the volumes of fuel consumption, CO and NOx emissions are relatively low because of low traffic volume. Fuel consumption and CO emissions concentrate on sub-arterial roads such as SPL Road (Shipailing Road), branch roads in the residential area in the southwest of the study area and east segment of XC Road (Xiongchu Road), while elevated levels of NOx emissions are identified in expressways and arterial roads, such as LY Road (Luoyu Road), a west segment of XC Road (near the gas station), LS south Road (Luoshi South Road) and ZS Road (Zhongshan Road).

During morning rush hours 8:00–10:00, fuel consumption and emissions increase across the study area. Elevated levels of fuel consumption and CO emissions shift from sub-arterial roads and branch roads to arterial roads and expressways such as LY Road, WL Road (Wuluo Road) and ZS Road. Besides, the high traffic volumes in morning rush hours and congestions in arterial roads lead to concentrations of CO and NOx emissions at the intersection of LY Road and LS South Road, and the intersection of ZN Road (Zhongnan Road) and WL Road. Elevated levels of NOx emissions remain on the same roads while the ranges have expanded.

During 14:00–16:00, CO emissions and fuel consumption increase in roads of low grade compared with that in 8:00–10:00. During evening hours 20:00–22:00, sub-arterial roads and branch roads in residential areas in the southwest of the study area further decrease compared with that in daytime. Because the number of taxis increase after evening rush hours, elevated levels of fuel consumption and emissions are identified in arterial roads such as WL Road, LY Road, intersection of LY Road and LS South Road, intersection of ZN Road and WL Road, ZS Road, Wuchang Railway Station and east segment of XC Road.

### 3.3. Analyzing Activity Types Producing Fuel Consumption and Emissions in the Study Area

**Figure 12:**
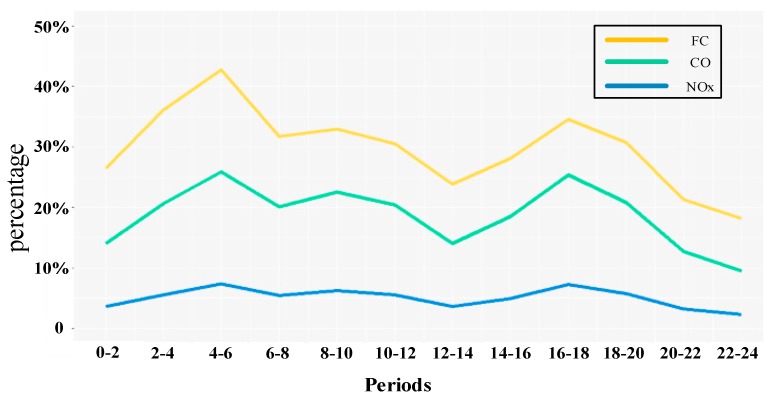
Percentages of fuel consumption/emissions of SAs with engine-on.

The visualization results in Figure 11 demonstrate that the proposed approach can discover the space-time distributions of fuel consumption and emissions intuitively and effectively. Furthermore, Figure 11 shows that not only arterial roads but also some of the small roads in residential areas such as southwest of the study area present high volume of fuel consumption and emissions, which is against our experience. From the analysis above, we know that both *MA*s and *SA*s with engine-on contribute to the fuel consumption and emissions. Therefore, we analyze and distinguish the types of fuel consumption and emissions of each road, and explore the patterns and mechanisms of fuel consumption/emissions in the study area. Figure 12 shows the percentages of fuel consumption/ emissions produced by *SA*s with engine-on in the study area.

**Figure 13:**
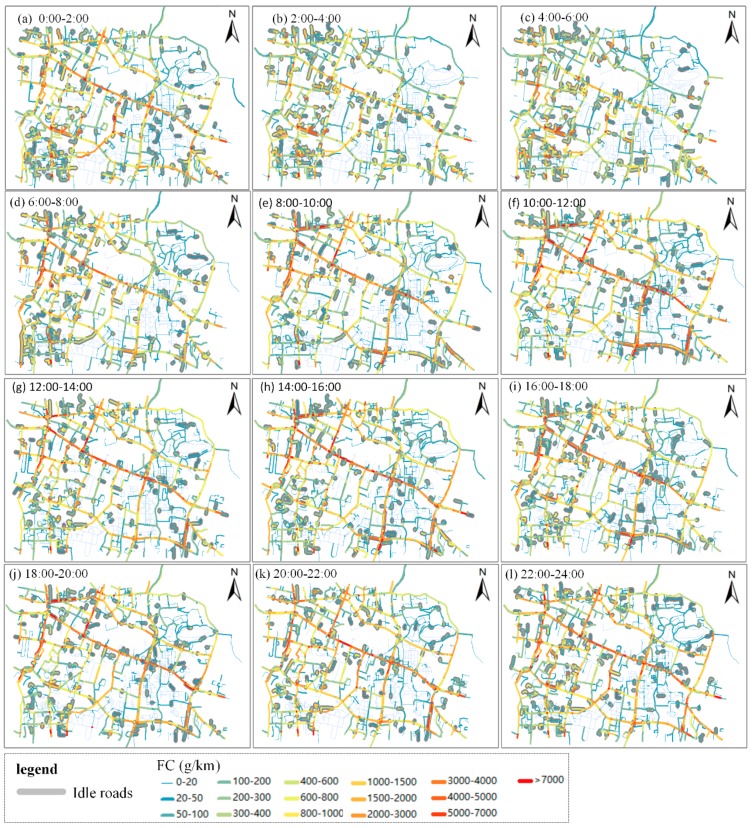
Space-time distribution of fuel consumption and idle roads in the study area in different period. (a) is 0:00–2:00, (b) is 2:00–4:00, (c) is 4:00–6:00, (d) is 6:00–8:00, (e) is 8:00–10:00, (f) is 10:00–12:00, (g) is 12:00–14:00, (h) is 14:00–16:00, (i) is 16:00–18:00, (j) is 18:00–20:00, (k) is 20:00–22:00, (l) is 22:00–24:00.

In Figure 12, the percentages of fuel consumption and emissions from *SA*s with engine-on in the 12 periods present a bimodal distribution. The first peak value appears during 4:00–6:00, in which fuel consumption, CO and NOx emissions of *SA*s with engine-on constitute for 42%, 25% and 5% of the whole fuel consumption and emissions in the study area. The other peak values are in evening peak hours (16:00–18:00), in which fuel consumption, CO and NOx emissions of *SA*s with engine-on constitute for 35%, 25% and 6% of the whole fuel consumption and emissions. The second peak value of fuel consumption is lower than the first peak values, and are nearly equal to the percentages in morning peak hours (8:00–10:00). Figure 12 also illustrates that *SA*s with engine-on have the strongest influence on fuel consumption compared with CO and NOx emissions. A large percentage of fuel consumption produced by *SA*s with engine-on on a road shows that speeds of taxis on the road approach to zero, implying there are traffic congestions or other stop behaviors with engine-on of taxis. In order to uncover the spatial-temporal distribution of fuel consumption produced by *SA*s with engine-on, we identify the roads where more than 70% of fuel consumption are produced by *SA*s with engine-on in the study area. Because the fuel consumption from the identified roads are mostly produced by vehicles under idling conditions, we name these identified roads as idle roads. Space-time distributions of fuel consumption and idle roads in the study area shown in Figure 13, where the grey-shaded roads are idle roads.

Figure 13 uncovers the space-time distribution patterns of idle roads and fuel consumption in the study area. In general, hot spots of idle roads tend to appear in arterial roads in the daytime while tend to appear in branch roads and residential areas at night and early-morning. In addition, the middle of ZS Road is identified as a hotspot of idle segments across a day because railway station locates in ZS Road. Taxis could stop or hover idly around railway station for waiting or picking-up/dropping-off passengers around the railway station, producing high idle fuel consumptions.

More specifically, during periods 0:00–6:00, there are fuel consumptions in non-idle arterial roads such as LY road, LS roads, indicating that the fuel consumptions in these arterial roads are produced from taxis’ normal running. Clusters of idle roads are identified in branches of arterial roads such as branches of LY Road, branch roads in the residential area in the southwest of the study area, and gas station in the west of XC Road. These branch roads have relatively higher fuel consumption than other roads in the period, indicating the fuel consumption in the periods are mainly caused by taxis’ hovering and refueling activities. 

During morning rush hours (period 4, 6:00–8:00), idle roads distribute more balanced compared with last periods. Fuel consumptions in arterial roads such as LN Road, ZS Road increase significantly, and idle roads in branch roads and gas stations decrease. During 6 a.m.–4 p.m., idle roads in branch roads in residential area decrease, while idle roads appear in arterial roads and intersections, such as LY Road, XC Road, LS Road, intersection of ZDQ Road (Zhuodaoquan Road) and XC Road, intersection of LS Road and LY Road, and intersection of LY Road and ZN Road. The characteristics of idle roads in daytime reflect that increasing traffic volumes in daytime lead to traffic congestions in arterial roads and intersections, bringing high idle fuel consumptions.

During evening rush hours (16:00–18:00), idle roads in branch roads and gas stations further decrease. In comparison, idle roads in arterial roads and sub-arterial roads increase, such as LY Road, WL Road, BY Road (Bayi Road) and MZ Road (Minzhu Road). Besides, idle range in XC Road expand. After 18:00, clusters of idle segments in arterial roads spread to sub-arterial roads, branch roads and gas stations.

## 4. Discussion

In this case study, we illustrate the effectiveness of the proposed methodology, which may be replicated in other cities throughout the world. Useful insights about fuel consumption/emissions estimation are uncovered through activity analysis with big data. First, we recorded the fuel consumption of an experimental vehicle (3.0 L) for verifying the estimating accuracy of the proposed approach. Estimation accuracy is significantly improved by considering activity types when compared with previous studies. Second, spatial-temporal distribution of fuel consumption/ emissions of taxis (1.4–2.0 L) in a network area shows that elevated levels of fuel consumption and emissions are identified on arterial roads with high traffic volume during the day and evening hours but on small roads in the early morning. We then explore the patterns and mechanisms of fuel consumption/emissions in the study area by analyzing the types of fuel consumption and emissions of each road. Our results indicate that the spatial-temporal patterns of fuel consumption of taxis are highly related to their activities, especially idling stops, which have a notable impact on fuel consumption. A major crisis is the “congestion” due to the number of vehicles on road, which is more severe during the rush hours. Besides, taxis’ idling stops (SAs with engine-on) that lead to high volumes of fuel consumption include not only traffic congestions but also non-service stop activities such as refueling or waiting for passengers. Hence, encouraging taxis to turn their engines off during their non-service stop activities in the elevated areas of idle roads can help to reduce the fuel consumption. Therefore, studying the patterns of taxis’ activities provide an understanding of the volumes and constitution of fuel consumption/emissions in urban traffic, and may bring solutions to the problem of fuel consumption.

In the case study, we validate the estimation methods with an experimental vehicle of 3.0 L capacity, while the accuracy of fuel consumption estimation in the experiment of a large area is unavailable because the real value of fuel consumption and emissions of the taxis (1.4–2.0 L) can hardly be obtained. For the same reason, many existing studies that estimate emissions/fuel consumption using GPS data only present the estimation results without quantitatively assessing their validity [2,10,11,12,13,14,15,16,17]. Although this study only validates the accuracy of fuel consumption estimation of 3.0 L gasoline vehicle, the estimations for the 3.0 L gasoline vehicle and 1.4–2.0 L gasoline vehicles are both based on the COPERT model using the same estimation principle and processing. Furthermore, the purpose of this study is to improve the estimations of fuel consumption and emissions by considering different types of activities. In both cases the fuel consumption and emissions are estimated in the same way, as a result, the validity of the 3.0 L vehicle is considered representative in this study.

While previous studies have not identified *MA*s and *SA*s in vehicles’ traces, the analysis in this study illustrates that in addition to different types of activities, modes of *SAs* also have a great impact on the fuel consumption/emissions produced during and after *SAs*. First, during *SAs* with engine-off, vehicles do not consume fuel or release emissions, but during *SA*s with engine-on, vehicles need to consume fuel and thus release emissions to keep the engines running. Second, because cold start emissions are produced when a vehicle restarts, they are closely related to the modes of *SA*. After an *SA* with engine-on, no cold start emissions are released, but after an *SA* with engine-off during which the temperature of the vehicle’s engine has decreased, cold start emissions are produced until the temperature of the engine has reached the stable temperature. Without analyzing the modes of *SA*s, cold start emissions in a vehicle’s trajectory cannot be estimated, which would lead to underestimations of fuel consumption/emissions since cold start emissions are many times higher than hot emissions. By comparison, this study identified *MA*s, *SA*s with engine-on and *SA*s with engine-off. Fuel consumption, hot emissions and cold start emissions are estimated according to different activity types, thus producing a more fine-grained and more precise estimation.

Lastly, besides bringing about deep insights about mechanisms of traffic fuel consumption/emissions, activity analysis with GPS big data also improves the accuracy of fuel consumption/emissions estimation. Compared with previous studies such as researches [14] and [17], we improved the accuracy and detailed level of fuel consumption/emissions estimation in two aspects. First, researches [14] and [17] divide the study areas into grids for analyzing the spatial distributions of emissions. In that way, moving parameters of vehicles are calculated based on Euclidean distances between GPS records, which leads to bias in the estimation of fuel consumption/emissions because vehicles actually move on the road network and run network distances rather than Euclidean distances. Moreover, emissions and fuel consumption are also visualized in units of planar spatial grids. Since a grid may contain several roads in the study area, patterns of fuel consumption/emissions in different roads cannot be distinguished. In contrast, we estimate and visualize fuel consumption/emissions based on road segments. With GPS trace matched to the road, we can restore the routes of vehicles and obtain more accurate moving parameters of vehicles from network distance, which is a critical factor in fuel consumption/emissions estimation.

## 5. Conclusions

The widespread spatial big data such as GPS data has brought fuel consumption/emissions estimation to a new perspective. While many researchers focus on the possibilities to understand the spatial-temporal distribution of fuel consumption/emissions with such big data, inadequate attention is paid to the estimation accuracy, which is the basis for uncovering the fuel consumption/emissions patterns. Emissions and fuel consumption are complex processes, related to factors including fuel parameters, vehicle model, and weather. In addition, fuel consumption/emissions are significantly affected by vehicles’ activities. Existing studies do not take the activity types of vehicles into consideration in estimation. Therefore, this study proposes approaches for estimating fuel consumption/emissions based on analysis of different types of activities in vehicles’ space-time paths. In our approach, we firstly build space-time paths of individual vehicles and extract moving parameters of each STPS. Then we present an N-dimensional representation approach for estimating and visualizing fuel consumption/emissions of both individual vehicle and a network area. In the case study, fuel consumption and emissions of a single vehicle and a road network area are estimated and visualized with GPS trace data. The estimating result illustrates that the estimating accuracy is significantly improved (88.6%) compared with the other two estimating approaches (71.02% and 78.2%) that do not distinguish activity types in estimation. The results indicate that the STPS based approach this study developed can estimate fuel consumption more accurately. In conclusion, the contributions of this article are mainly in the following aspects.

First, we present an N-dimensional representation framework for estimating and visualizing fuel consumption/emissions. Quantities and distribution patterns of fuel consumption/emissions can be explored under the framework.

Second, we determine different types of activities from space-time paths and identify mobile activities (*MA*s), stationary activities (*SA*s) with engine-on and with engine-off. Fuel consumption, hot emissions, and cold start emissions are estimated according to different activity types, thus producing a more fine-grained and more precise estimation.

Finally, based on the determination of different types of activities, we further analyzed the constituents of fuel consumption of each road, that is, what percentage of the whole fuel consumption do *MA*s and *SA*s with engine-on contribute. As a result, patterns and mechanisms of fuel consumption in the study area can be uncovered.

The limitations of this study include: (1) this study provides a methodology that estimates fuel consumption/emissions based on activity types. The study area is considered sufficient for evaluating the effectiveness of the proposed method, while citywide distribution patterns of fuel consumption/emissions are not reflected in this study. (2) The spatiotemporal patterns of fuel consumption/emissions during a day are uncovered in this study, while the differences between workdays and weekends are not included. (3) This study mainly puts effort on analyzing *SA*s in taxis’ GPS traces, but patterns of vehicles’ *MA*s are not analyzed in-depth. Future studies include estimating fuel consumption/emissions in a larger scale and a larger time span and studying approaches to reduce fuel consumption/emissions of taxis by adjusting their activity patterns, including *SA* patterns and *MA* patterns.

## Figures and Tables

**Table 1:** Data and models in recent studies.

Models	Resolution of GPS Data		Emission Model		Driving Modes Analysis	Stationary Activity Analysis	Hot/Cold Start Emissions Estimation
	High	Low	Macro	Micro			
Gühnemann et al. [11]		√	√				
Shang et al. [14]		√	√				
Luo et al. [17]		√	√				
Zhao et al. [15]		√		√			
Sun et al. [13]	√			√	√		
Nikoleris et al. [18]	√			√	√		
Nyhan et al. [2]	√			√	√		
This study		√	√			√	√

**Table 2:** Description of GPS data of a gasoline experimental vehicle from 23 March to 25 March 2016.

VID	Time	Longitude	Latitude
1	2016-03-23 8:00:00	114.3550441	30.5604297
1	2016-03-23 8:00:09	114.3550441	30.5604297
1	2016-03-23 8:00:20	114.3549372	30.5601104
1	2016-03-25 17:59:50	114.35481	30.559673
1	2015-03-25 18:00:01	114.3550441	30.5604297

**Table 3:** Vehicle and fuel parameters of the experimental vehicle and real fuel consumption.

**Vehicle Parameter**	**Model**	**Year**	**Engine Capacity**
	Buick Park Avenue	2009	3.0 L
**Fuel Parameter**	**Gasoline label Number**	**Density**	
	No. 97	0.737 g/mL	
**Real Fuel Consumption**		40.66 kg	

**Table 4:** Description of GPS data of taxi GPS data in Wuhan, on 6 May 2015.

VID	Time	Longitude	Latitude	Direction	Speed	Status
1001	2015-05-06 00:00:00	114.260889	30.583315	265	20	0
1001	2015-05-06 00:01:01	114.260765	30.583395	190	40	0
1001	2015-05-06 00:02:00	114.260765	30.583415	188	19	0
1002	2015-05-06 08:01:02	114.26122	30.583289	120	10	1
1002	2015-05-06 08:02:00	114.26089	30.583322	30	29	1

**Table 5:** Parameters of emission factors for different pollutants in COPERT model.

Pollutants	a	b	c	d	e
CO	71.7	35.4	11.4	−0.248	0
NO_x_	9.29 × 10^−2^	−1.22 × 10^−2^	−1.49 × 10^−3^	3.97 × 10^−5^	6.53 × 10^−6^
Hydrocarbon	5.57 × 10^−2^	3.65 × 10^−2^	−1.1 × 10^−3^	−1.88 × 10^−4^	1.25 × 10^−5^

**Table 6:** Functions for cold start emissions estimation per start.

Pollutants	*f*(*T*, *V*)	*h*(*ş*)	*g*(*t*)
CO	4.291 − 0.176*T + 0.012*V*	(1 − e^−7.288*δ*^)/(1 − e^−7.288^)	(1) 4.614 × 10^−3^ *t* − 2.302 × 10^−6^ *t*^2^ − 2.966 × 10^−9^ *t*^3^ (*t* ≤ 720 min) (2) 1 (*t* > 720 min)
HC	9.093 − 0.459*T + 0.054*V*	(1 − e^−8.624*δ*^)/(1 − e^−8.624^)	(1) 7.641 × 10^−3^ *t*^−2^ − 2.639 × 10^−5^ *t*^2^ + 3.128 × 10^−8^ *t*^3^ (*t* ≤ 240 min) (2) 0.625 + 5.208 × 10^−4^ *t* (240 ≤ *t* ≤ 720 min) (3) 1 (*t* > 720 min)
NOx	0.808 − 0.005*T + 0.015*V*	(1 − e^−0.739*δ*^)/(1 − e^−0.739^)	(1) 7.141 × 10^−3^ *t* + 1.568 × 10^−3^ *t*^2^ − 3.204 × 10^−5^ *t*^3^ + 1.594 × 10^−7^ *t*^4^ (*t* ≤ 50 min) (2) 1.290 − 4.030 × 10^−4^ *t* (50 ≤ *t* ≤ 720 min) (3) 1 (*t* > 720 min)

**Table 7:** Comparison of fuel consumption estimation results of average speed based approach and STPS-based approach in this article.

Estimating Approaches	Average Speed (km/h)	Fuel Consumption Factor (g/km)	Travel Distance (km)	Fuel Consumption Estimated (kg)	Real Fuel Consumption (kg)	Accuracy
Average speed of a vehicle [11]	22.23	116.64	490.84	57.25	40.66	71.02%
Average speed of trace points [13]	Depend on GPS trace points			31.79		78.2%
Proposed STPS-based approach	Depend on each STPS			45.31		88.6%
